# Application of Hyperspectral Technology with Machine Learning for Brix Detection of Pastry Pears

**DOI:** 10.3390/plants13081163

**Published:** 2024-04-22

**Authors:** Hongkun Ouyang, Lingling Tang, Jinglong Ma, Tao Pang

**Affiliations:** College of Mechanical and Electrical Engineering, Sichuan Agriculture University, Ya’an 625000, China; 2022317025@stu.sicau.edu.cn (L.T.); 202106317@stu.sicau.edu.cn (J.M.)

**Keywords:** hyperspectral imaging, crisp pear, sugar content, machine learning

## Abstract

Sugar content is an essential indicator for evaluating crisp pear quality and categorization, being used for fruit quality identification and market sales prediction. In this study, we paired a support vector machine (SVM) algorithm with genetic algorithm optimization to reliably estimate the sugar content in crisp pears. We evaluated the spectral data and actual sugar content in crisp pears, then applied three preprocessing methods to the spectral data: standard normal variable transformation (SNV), multivariate scattering correction (MSC), and convolution smoothing (SG). Support vector regression (SVR) models were built using processing approaches. According to the findings, the SVM model preprocessed with convolution smoothing (SG) was the most accurate, with a correlation coefficient 0.0742 higher than that of the raw spectral data. Based on this finding, we used competitive adaptive reweighting (CARS) and the continuous projection algorithm (SPA) to select key representative wavelengths from the spectral data. Finally, we used the retrieved characteristic wavelength data to create a support vector machine model (GASVR) that was genetically tuned. The correlation coefficient of the SG–GASVR model in the prediction set was higher by 0.0321 and the root mean square prediction error (RMSEP) was lower by 0.0267 compared with those of the SG–SVR model. The SG–CARS–GASVR model had the highest correlation coefficient, at 0.8992. In conclusion, the developed SG–CARS–GASVR model provides a reliable method for detecting the sugar content in crisp pear using hyperspectral technology, thereby increasing the accuracy and efficiency of the quality assessment of crisp pear.

## 1. Introduction

Pears are one of the world’s most popular fruits [1]. Pears, compared to other fruits, have a higher dietary fiber content and can have more favorable effects on the human gastrointestinal tract, making them popular among consumers [2].

The sugar content in pears can influence their flavor, so it is an essential predictor of pear quality. The pear quality control procedure in many countries now involves the detection of sugar content [3]. Currently, the quality inspection and classification of most fruits primarily rely on manual examination [4], which is subjective and inefficient [5]. Now, the quality features of most fruit can be directly detected, exhibited, and identified using recent advancements in computer vision technology such as RGB and hyperspectral images. Additionally, optical imaging technologies such as spectral imaging are becoming increasingly popular with the development of hyperspectral sensors for the automated detection and nondestructive grading of fruit quality [6]. These systems can be used to collect a large amount of digital data related to fruit properties [7]. When processing large batches of fruit-grading activities, the fruit quality detection accuracy and detection time of contemporary optical imaging technology are higher and lower, respectively, than those of previous approaches [8]. Notably, the application of contemporary optical imaging equipment for nondestructive testing can substantially reduce labor costs while increasing testing efficiency.

Hyperspectral imaging techniques can be used to efficiently capture internal fruit quality information, as differences in fruit quality are reflected in differences in waveband and spatial distribution information [9]. These methods have performed well in testing the fruit quality, accurately detecting the attributes of several fruits, including glucose content [10], persimmon skin hardness [11], banana water content [12], strawberry skin abrasions [13], and citrus maturity [14]. Gamal El Masry et al. employed hyperspectral imaging technology in the visible and near-infrared (400–1000 nm) regions in 2006 to build a model to nondestructively quantify indicators such as the total soluble solid (TSS) content in strawberries [15]. In 2016, Jiangbo Li et al. applied long-wave near-infrared hyperspectral imaging technology to evaluate the soluble solid content (SSC) in pears [16]. Dongyan Zhang et al. used hyperspectral imaging technology to quantify the sugar content in a specific pear variety (Danshan) in 2018 [17]. In 2023, Min Xu et al. used hyperspectral technology with the deep-learning-based stacked autoencoder (SAE) method to construct a deep learning model to quickly detect the TSS in Kyoho grapes. As such, nondestructive testing (NDT) [18] represents a suite of analytical techniques employed for the evaluation of a material’s properties without causing damage.

Most scholars have chosen to screen features from several bands in hyperspectral data to find characteristic bands that are strongly associated with fruit quality. These methods can efficiently handle some of the features that may be included in the whole spectrum when combined with hyperspectral imaging technology. In these models, various wavelengths can cause a number of issues such as collinearity, redundancy, and noise interference [19]. To address these issues, the feature extraction algorithm and the quality of the extracted features can be enhanced to improve the model’s prediction performance [20]. A variety of variable selection algorithms have been created for feature extraction technology to generate parsimonious models. Nogaard et al. developed interval partial least squares (iPLS), a graph-oriented local modeling approach, which they tested on a near-infrared (NIR) spectral dataset based on 60 beer samples. The spectral correlation coefficient was calculated: the root mean square error was 0.17%, which was four times less than that of the whole spectrum [21]. Munera investigated the interior quality of persimmons using hyperspectral imaging technology and predicted hardness using the continuous projection and partial least squares regression models, achieving an Rp2 prediction accuracy of 0.80 and an RMSEP of 3.66 [11]. Choi et al. employed typical normal transformation and smooth convolution preprocessing together with the partial least squares regression approach to develop a model using the near-infrared spectroscopy data of pear sugar concentration. The correlation coefficient on the prediction set ranged between 0.90 and 0.96; the root mean square error ranged from 0.29 to 0.33 [22]. Although these variable selection algorithms are novel, their algorithm fusion techniques are straightforward. The algorithm is neither tuned nor preprocessed step-by-step, limiting further increases in model detection performance [9].

In this study, we aimed to address the aforementioned issues by developing a method combining feature extraction engineering methodologies. We used a genetic algorithm (GA) to enhance the support vector machine algorithm (SVR), which was then paired with three preprocessing methods: standard normal transformation (SNV), multivariate scattering correction (MSC), and smooth convolution (SG), as well as competitive adaptive reweighting. The competitive adaptive reweighting (CARS) algorithm and the continuous projection algorithm (SPA) were the two feature extraction approaches. Using hyperspectral data, we developed a model for detecting the sugar content in crisp pear, offering both theoretical reference and technological assistance for the grading and nondestructive testing of crispy pear quality. The following are the novel features of this study:

(1) A crisp pear sugar content dataset was developed and published using hyperspectral imaging technology;
(2) The feasibility and ideal model of the optimized genetic algorithm were investigated for predicting the sugar content in pear.

## 2. Related Studies

### 2.1. Hyperspectral Technology

Spectroscopy is an interdisciplinary study of physics and chemistry, examining the interaction of electromagnetic waves with substances in a spectrum [23]. Because the atoms that comprise each substance have distinct spectral lines, spectra can be used to identify substances and determine their chemical compositions [24]. This is known as spectral analysis. Hyperspectral imaging technology has the characteristics of both traditional imaging and spectral analysis and can be used to simultaneously obtain the spatial and spectral information of a detected object, as well as to detect physiological characteristics of, for example, fruit, via detecting light absorption, transmission, and reflection [25]. For example, hyperspectral imaging technology has been used to identify early fruit rot [26] and estimate strawberry moisture content and maturity [27].

### 2.2. Genetic Algorithm (GA)

The evolutionary principles of nature inspired the genetic algorithm (GA). The GA is a search technique used for discovering optimal solutions that is applied in a variety of optimization problems [28], for example, studying multiobjective optimization models for a sustainable agricultural industry structure [29] and the optimization of apple disease segmentation and classification based on strong correlation and feature selection [30]. As such, the GA was used in this study to optimize the SVM model generated using the spectral data from crisp pear obtained using hyperspectral technology to accurately identify the sugar content in crisp pear.

### 2.3. Support Vector Machine (SVM)

A support vector machine (SVM) is a binary generalized linear classifier that uses supervised learning to classify data that performs particularly well when dealing with small sample sizes and in nonlinear and high-dimensional situations [31]. An SVM is currently capable of handling multiclassification problems and performing application tasks in agricultural detection owing to extensive research and development; for example, see [32] for the application of a support vector machine in precision agriculture and [33] for its application in the multicategory recognition of maize seedlings/weeds in visible/near-infrared imagery.

## 3. Materials and Methods

### 3.1. Data Production

**Figure 1:**
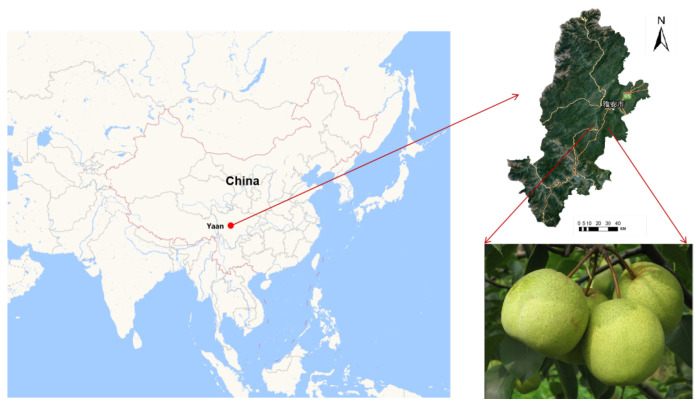
A total of 168 crisp pear samples were collected in November 2022, in Ya’an City, Sichuan Province, China (29.9890 latitude north, 102.9820 longitude east).

The sample collection area is shown in Figure 1. A total of 168 crisp pear samples were collected in November 2022, in Yucheng District, Ya’an City, Sichuan Province (29.9890 latitude north, 102.9820 longitude east). The samples were consistent in terms of size, surface integrity, and maturity. The experiment began with meticulous wiping and random numbering of each pear sample. Then, each sample was placed in the experimental setting for 24 h to guarantee that the sample’s temperature was synchronized with the ambient temperature, establishing the groundwork for later detection work.

**Figure 2:**
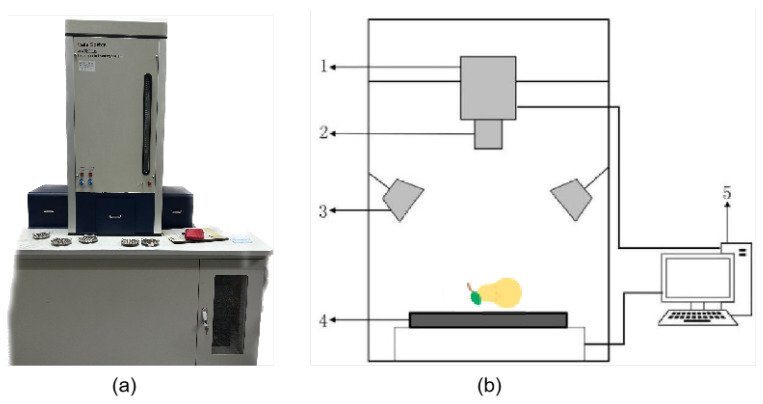
(**a**) Hyperspectral sorter; (**b**) structural schematic diagram of hyperspectral system: 1. CCD camera; 2. spectrometer; 3. diffuse reflection light source; 4. electric translation stage; 5. computer.

Figure 1 shows the specific geographical location of the study, as well as the specific origin of the pear samples. The Gaia Sorter hyperspectral sorter, manufactured by Beijing Zhuoli Hanguang Company, was employed to gather data, as illustrated in Figure 2a. The system included a high-resolution CCD camera (1344 × 1024 pixels), a spectrometer (Image-Spectral Image, working wavelength range of 387 nm to 1034 nm, capable of collecting spectral information in 256 wavelength bands), a diffuse reflection light source (primarily a bromine tungsten lamp with a power of 200 W), an electric translation stage, and a computer system. All acquisition activities were performed in a specialized dark box, as illustrated in Figure 2b, to avoid the impact of external light on image acquisition.

We employed SpecView Version 3 (V3) software to precisely adjust the parameters of the instrument before collecting hyperspectral images of the samples to ensure the capture of high-quality images. These parameters included exposure time, spectral resolution, and the electronically controlled mobile platform’s action parameters. Furthermore, the instrument was warmed for 30 min before use to ensure constant temperature and light intensity during the experiment. Given the potential impact of ambient conditions and the instrument on hyperspectral images, the original images were also subjected to ordinary black-and-white corrective processing [34]. Equation (1) shows the adjustment formula:

(1) $$I=\frac{I_{0}-B}{W-B}$$

where *I* is the corrected image, $I_{0}$ is the original image, *B* is the black standard image, and *W* is the white standard image.

We set the camera exposure time to 11 ms, the distance between the camera objective lens and the platform to 190 mm, and the moving platform speeds to 0.5 cm/s and 1 cm/s. Furthermore, the camera’s spectral range was 387–1034 nm, with a spectral resolution of 2.8 nm.

The spectrometer’s imaging method was as follows. First, the spectrometer scanned each row of pixels in the sample to be measured to produce a single row of image and spectral information. Second, the electric translation stage advanced the sample along the predetermined path, successively exposing and imaging the placed CCDs in the longitudinal direction. As such, the comprehensive three-dimensional hyperspectral image data from the sample were acquired when paired with horizontal and vertical imaging information. Each crisp pear sample’s spectral data had a fixed 1344 × 1024-pixel resolution and included 256 wavelength bands. Given the equipment noise at both ends of the spectrum range, we selected 237 wavelength data for each sample in the 400–1000 nm range to analyze. Following the processing of these 168 pieces of data, they were separated into a training set (118 samples) and a prediction set (50 samples) in a 7:3 ratio. Figure 2 depicts the raw spectrum data. We revealed three distinct absorption valleys in the 0–100 nm to 200–300 nm wavelength ranges.

**Figure 3:**
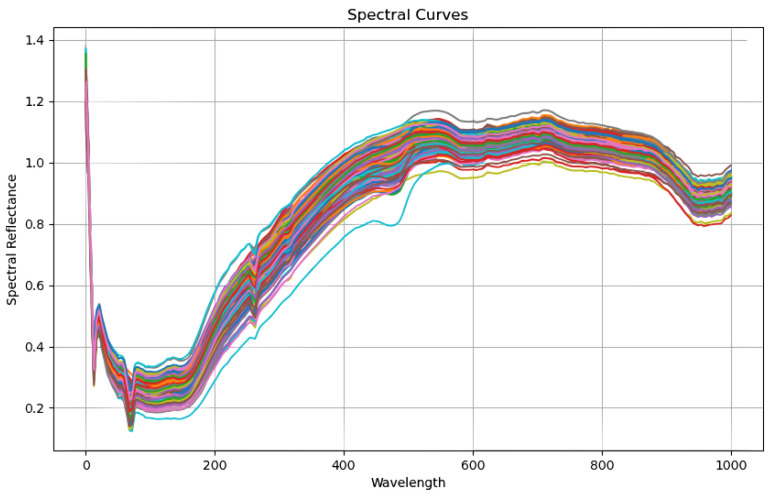
Raw spectral data.The curves of different colors represent the different wavelengths collected by different samples during data collection. The horizontal coordinate represents wavelength data and the vertical coordinate represents reflection data.

Figure 3 depicts the raw spectrum data. The spectral data of 168 unprocessed pear samples were collected. A Fanover digital sugar content refractometer was used in this study to determine the physical and chemical sugar contents in crisp pear. The device had a resolution of 0.1% and an accuracy of 0.2% for measuring fruits and vegetables with a sugar content of up to 32%. It performed steadily at ambient temperatures ranging from 10 to 40 °C, allowing for the reliable detection of sugar content in our tests. The sugar content in 168 crisp pear samples was assessed by following the NY/T2637-2014 standard [35]. This refractometer was used to test sugar content in Brix units under a constant laboratory temperature of 19 °C. To assure the accuracy of the measurements, the refractometer’s measuring window was cleaned with distilled water and dried with special lens cleaning paper before each measurement. Then, we removed the pulp from the equatorial portion of the pear, squeezed off the juice, and placed it on the refractometer’s window. Each sample’s Brix value was measured three times independently, with the average serving as the final record. The 168 samples were divided into training and test sets in a 7:3 division during the experiment. Table 1 shows the division of the training and test sets prior to the formal processing of the spectral data, as well as the calculation of the parameters.

Table 1 shows the division of the training set and test set and the parameter calculation for each part before the formal processing of spectral data. The sugar level in the training set samples ranged from 7.4 to 12.5, as shown in Table 1, and that in the test set samples ranged from 8.4 to 10.5. The test set’s standard deviation, 1.52, was less than that in the training set of 2.83, indicating that the test set’s data distribution was more concentrated.

### 3.2. Data Preprocessing

The convolution smoothing (Savitzky–Golay, SG) algorithm is an accurate and efficient method for smoothing spectral data. This algorithm calculates the average value within the smoothing window through weighted least squares fitting, thereby highlighting the importance of the center point [36]. This method uses polynomials to perform the least squares fitting of spectral data to achieve data smoothing. This requires selecting a fixed-size window, treating all spectral data within the window as a whole, representing each measurement point $x=\left[\begin{array}{c} -m,1-m,\ldots ,0,1,\ldots m \end{array}\right]$, and using the polynomial formula shown in (2) to complete the fitting:

(2) $$p\left(x\right)=\sum_{k=0}^{N}a_{k}x^{k}$$

**Figure 4:**
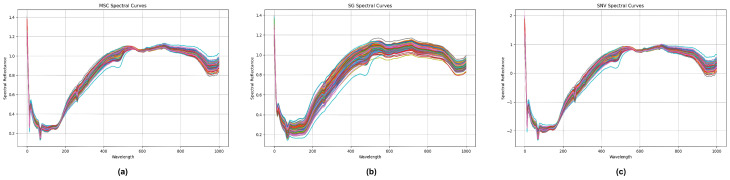
Comparison chart of processed hyperspectral data after (**a**) multivariate scattering correction, (**b**) convolution smoothing, and (**c**) standard normal transformation. The curves of different colors represent the different wavelengths collected by different samples during data collection. The horizontal coordinate represents wavelength data and the vertical coordinate represents reflection data.

The residual between the original spectrum and the fitted line was calculated, and its minimum value was used as the boundary point to solve the optimal coefficient matrix $B=X{\left(X^{T}X\right)}^{-1}X^{T}$. Then, this coefficient matrix was convolved with the spectral data from each sample, accurately smoothing the original spectral data. This not only ensured the integrity of the data but also strengthened the role of the center point in the entire dataset, providing a more accurate and stable basis for subsequent spectral analysis. In addition to the convolution smoothing algorithm, the standard normal transformation (SNV) and multivariate scattering correction (MSC) algorithms are common spectral data preprocessing algorithms. The three data preprocessing effects of standard normal transformation (SNV), multivariate scattering correction (MSC), and convolution smoothing (SG) are shown in Figure 4.

The trend of each color band in Figure 4 represents the variation in hyperspectral reflectance response for each experimental sample. Different color bands represent different experimental samples.

### 3.3. Feature Selection Method

The CARS methodology is based on the Monte Carlo sampling method, in which adaptive reweighted sampling (ARS) is the main technology. The main advantage of this algorithm is that it can effectively use the ARS strategy to select wavelength points with higher absolute regression coefficient values; then, this algorithm screens out the subset corresponding to the minimum error using cross-validation, thereby efficiently identifying the optimal variable combination [37]. During the CARS implementation step, some samples are randomly selected from the correction set for modeling, whereas the remaining samples are used as the prediction set. The number of samplings (N) must be determined ahead of time. The program then uses an exponential decay function to exclude wavelength points with fewer weighted regression coefficients. Each round of sampling uses the ARS approach to select wavelengths from the previous round’s variable collection. Here, we effectively produced N sets of candidate feature wavelength subsets and their accompanying attributes after N rounds of sampling. Finally, the characteristic wavelength is chosen from the subgroup with the lowest value. Each variable is given a weight by the CARS algorithm. The higher the weight, the larger the variable’s contribution to the model, and the probability of being selected proportionately increases. The specifics of this process’s computation can be found in Formulas (3) and (4):

(3) T=XW

(4) y=Tc+e=XWc+e=Xb+e

Here, *X* is a spectral matrix with M rows and P columns; *T* is the score matrix of *X*; *W* is a linear combination system of *X* and *T*; *C* is a regression coefficient vector representing the partial least squares model established with *T*; and *e* is the error vector. The weight ω is defined as follows:

(5) $$\omega_{i}=\frac{|b_{i}|}{\sum_{i=1}^{p}|b_{i}|}$$

When implementing the CARS algorithm, the number of Monte Carlo samplings N needs to be determined in advance. This algorithm relies on cross-validating each candidate variable subset and comparing their root mean square errors (RMSECVs) when selecting the optimal variable subset. Among these variable subsets, the subset with the smallest RMSECV is selected as the optimal variable subset. Importantly, the CARS algorithm eliminates uninformative or low-information variables by performing two key steps, exponential decay function (EDF) and adaptive reweighted sampling (ARS), during each round of running. Specifically, EDF defines the proportion of variables retained in each run, which is calculated as follows:

(6) $$r_{i}=ae^{-ki}$$

Under certain conditions, *a* and *k* are treated as constants. Specifically, in the first run, the wavelength used for modeling is the full wavelength, so $r_{1}=1$. By the *N*th run, two wavelengths are used for modeling, as shown in Equation (7), where constants *a* and *k* are defined in Formulas (8) and (9):

(7) $$r_{n}=\frac{2}{p}$$

(8) $$a={\left(\frac{P}{2}\right)}^{\frac{1}{\left(N-1\right)}}$$

(9) $$k=\frac{\ln\left(\frac{p}{2}\right)}{N-1}$$

In the process of wavelength selection, we first use the exponential decay function (EDF) to quickly eliminate those variables with lower weights. Second, using the adaptive reweighted sampling (ARS) method, according to the survival of the fittest principle, from the remaining $p\times r_{i}$, we select a new subset of variables. Third, the cross-validation method is used to calculate the root mean square error (RMSECV) of this new subset, which is used as a benchmark for the next round of iteration. This series of loop iterations is a continuous optimization based on the results of the previous round, aiming to gradually approach the optimal solution. This process not only ensures the stability and accuracy of the model but also increases the efficiency and practicality of the calculations.

### 3.4. Principle of Genetic Algorithm

The starting population in the genetic algorithm optimization method used in this study was composed of a series of solutions, each of which was represented by a chromosome and reflected a specific set of parameter configurations. The system then followed a sequence of selection, crossover, and mutation operations based on the defined fitness function, with the goal of screening and optimizing the individuals in the population. Individuals with good fitness were retained first, whereas those with low fitness were gradually phased out. This screening and optimization cycle continued until the specified termination conditions were met. Algorithm 1 depicts the detailed method of this algorithm.
**Algorithm 1** Genetic algorithm optimization    **Input:**
     Problem definition (including chromosome representation, fitness function, etc.)
     Population size: pop_size
     Crossover probability: crossover_rate
     Mutation probability: mutation_rate
     Maximum iterations: max_generations
    **Output:** Best chromosome or near-optimal chromosome
    **function** Initialize
     **return** [RandomChromosome() for _ in range(pop_size)]
    **end function**
    **function** SelectParents(pop)
     **return** two chromosomes based on fitness from pop
    **end function**
    **function** CrossoverAndMutate(p1, p2)
     **if** random()<crossover_rate **then**
      combine p1 and p2
     **end if**
      mutate resulting chromosomes with mutation_rate
     **return** two offspring
    **end function**
    **function** Execute
     population← Initialize
     **for** i←1 **to** max_generations **do**
      new_population←[]
      **for** j←1 **to** ⌊pop_size/2⌋ **do**
         parent1, parent2← SelectParents(*population*)
         new_population.extend(CrossoverAndMutate(*parent*1, *parent*2))
      **end for**
      population←new_population
     **end for**
     **return** highest fitness chromosome from population
    **end function**


The genetic algorithm’s selection operation step picks excellent individuals from the old population based on a given probability using tactics such as roulette selection, random competition selection, and best retention selection. A certain crossover operator causes partial portions of chromosomes to be transferred between two individuals, resulting in the generation of new chromosomes. The most common crossover methods used for this are single-point, two-point, and uniform crossover. The goal of the mutation operations is to develop individuals with improved performance by changing particular sections of individual chromosomes. The fitness function, as a vital signal for measuring individual performance, is critical in the selection operation and ensures the algorithm’s correctness and reliability. The use of the genetic algorithm increases not only the model’s convergence speed and effect but also the accuracy of detecting the sugar content in crisp pear.

### 3.5. Principle of Support Vector Machine Algorithm

**Figure 5:**
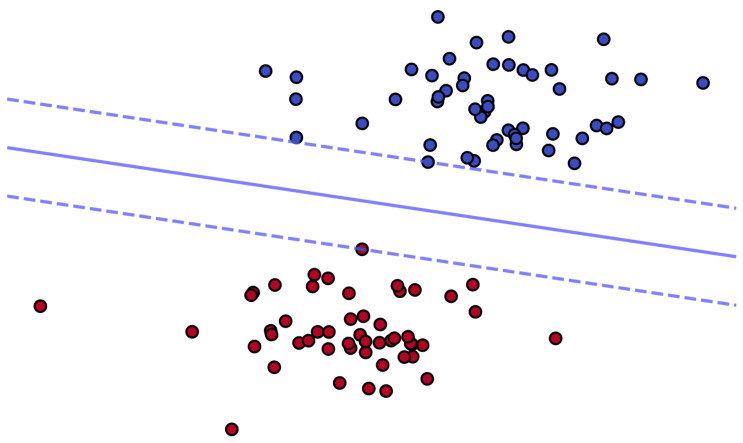
Support vector machine classification diagram. The solid line simulates the decision boundary that maximizes the classification interval of the data points, and the space between the two dashed lines represents the maximum confidence interval that exists under the decision boundary.

The support vector machine’s main purpose is finding a decision boundary that maximizes the classification interval, also known as the maximum margin hyperplane. Many hyperplanes, as illustrated in Figure 5, can be used to discriminate between two types of samples, but the ideal hyperplane is the one that minimizes the sum of the distances from all sample locations to the plane. This distance is referred to as the classification interval [31].

The derivative model, support vector regression (SVR), is an extension of SVM in regression prediction that has the goal of minimizing the total deviation between the predicted and actual values. The core of SVR lies in selecting an appropriate kernel function. We used the radial-basis kernel function (RBF) to build a prediction model. Differently from the discrete output of classification problems, the output of regression problems is continuous. For example, in this study, RBF was used to represent the predicted value of sugar content in pears. Unlike the classification problem, where the outputs “1” and “2” indicate the intact and bruised pear status, respectively, the output in the regression problem is continuous, which reflected the sugar content in the pears in this study. In addition, when processing spectral data, considering linear and nonlinear conditions, the SVR model adopts different regression function formulas, that is, Formula (10) under linear conditions and Formula (11) under nonlinear conditions, to achieve more accurate predictions. Under nonlinear conditions, kernel functions *x* and Lagrange multipliers $alpha_{i}$ and $alpha_{i}^{*}$ are all key parameters and require careful adjustment and inspection to ensure the best model performance.

(10) $$f\left(x\right)=\sum_{i-1}^{n}\left(\alpha_{i}-\alpha_{i}^{*}\right)<x_{i}$$

(11) $$f\left(x\right)=\sum_{i=1}^{n}\left(\alpha_{i}-\alpha_{i}^{*}\right)k<x_{i}$$

In summary, by creatively combining SVM and SVR, we predicted the sugar content in crisp pear, thus providing a reliable technique for assessing the quality of this fruit.

### 3.6. Improved Support Vector Machine Based on Genetic Algorithm

**Figure 6:**
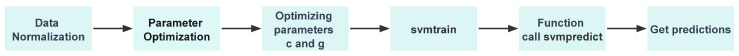
Implementation flow chart of SVM algorithm based on genetic algorithm optimization.

The parameter choices of the radial-basis function (RBF), particularly the kernel function parameter (g) and penalty factor (C), play an important role in the application of a support vector machine (SVM) [38]. The kernel function parameter (g) directly impacts the model’s generalization ability: a larger (g) value leads to the model being excessively complex and impairs prediction accuracy for unknown samples. This is typically observed as overfitting. Conversely, lower (g) values may result in the model being undertrained, i.e., underfitting. Similarly, the penalty factor (C) substantially impacts model performance: a larger (C) value limits the tolerance for training errors and raises the risk of overfitting, whereas a lower (C) value may impair the model’s general performance, leading to underfitting. As a result, to attain the most accurate SVM prediction performance, these two parameters must be precisely set. For this purpose, a genetic algorithm was used in this study to optimize the SVM parameters g and C and to develop a prediction model that accepts crisp pear sugar content spectral data as the input and crisp pear sugar content prediction value as the output. The model was developed and evaluated in the MATLAB R2023a environment, and the RBF kernel function was used as the SVM kernel parameter. Figure 6 shows the MATLAB implementation process for SVM and its optimization method.

In this study, the genetic algorithm’s optimization was designed to minimize the error rate of the support vector machine (SVM), with the error rate serving as the fitness function. The procedure started with a population that is randomly generated, with each population member representing a set of hyperparameter values. The error rate for each population member was calculated by training and testing the SVM model, and this error rate was used as the fitness value. We continued to optimize the hyperparameters C and g by selecting the individual with the highest fitness as the parent and establishing a new-generation population through crossover and mutation procedures. This genetic algorithm iterated until either the set number of iterations or the fitness criterion was met. Finally, the algorithm returned the optimum parameter values [39], which were used to build the SVM model to increase prediction accuracy and dependability [40].

## 4. Experimental Results

### 4.1. Evaluation Indicators

We used the Pearson correlation coefficient (r) squared, root mean square error RMSE (root mean square error), modeling determination coefficient of the training set and prediction set of the prediction model $R_{c}^{2}$, verification coefficient of determination $R_{p}^{2}$, and root mean square error of the correction and prediction sets to evaluate the development model for predicting crisp pear sugar content RMSEC. RMSEP [41] reflects the fitting effect of the predicted values with the true values in the model training and prediction sets. The value of the coefficient of determination ranges between zero and one, where, the closer the value to one, the higher the accuracy of the prediction model and the better the fitting degree. RMSEC and RMSEP reflect the degree of deviation between the predicted values for the samples in the training and prediction sets and the true values. The closer the value is to zero, the smaller the deviation of the model prediction value and the higher the inversion accuracy [42]. The calculation formulas are as follows:

(12) $$RMSEC=\sqrt{\frac{1}{n_{c}}\sum_{i=1}^{n_{c}}{\left[Y_{t}\left(i\right)-Y_{c}\left(i\right)\right]}^{2}}$$

(13) $$RMSEP=\sqrt{\frac{1}{n_{\nu }}\sum_{i=1}^{n_{\nu }}{\left[Y_{t}\left(i\right)-Y_{\nu }\left(i\right)\right]}^{2}}$$

(14) $$R_{c}^{2}=1-\frac{\sum_{i=1}^{n_{c}}{\left[Y_{t}\left(i\right)-Y_{c}\left(i\right)\right]}^{2}}{\sum_{i=1}^{n_{c}}{\left[Y_{t}\left(i\right)-Y_{m}\right]}^{2}}$$

(15) $$R_{p}^{2}=1-\frac{\sum_{i=1}^{n_{v}}{\left[Y_{t}\left(i\right)-Y_{\nu }\left(i\right)\right]}^{2}}{\sum_{i=1}^{n_{v}}{\left[Y_{t}\left(i\right)-Y_{m}\right]}^{2}}$$

where $n_{c}$ and $n_{v}$ are the number of samples in the crisp pear correction set and prediction set, respectively; $Y_{t}\left(i\right)$ is the true measured value of the ith sample; $Y_{c}\left(i\right)$ and $Y_{v}\left(i\right)$ are the predicted values of the samples in the crisp pear correction and prediction sets, respectively; and *i* and $Y_{m}$ and are the correction and prediction sets, respectively, which are the averages of real measurements.

### 4.2. Basic Experiments and Settings

**Figure 7:**
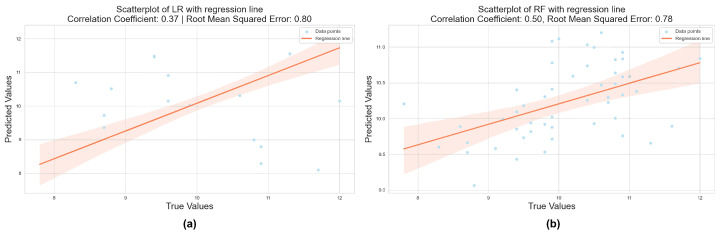
Prediction results of the linear regression model (**a**) and random forest model (**b**), respectively. The shaded part in red indicates the error range for fitting data points under this curve.

We first selected two classic basic regression approaches, linear regression and random forest, to further investigate the performance of the proposed optimization model in tests. Both techniques have demonstrated consistent and outstanding performance in a range of settings [43]. As a result, we argued that starting from their performance might provide a beneficial reference for later optimization of performance. As a result, we first compared these two fundamental methodologies. The data were randomly divided in a 7:3 ratio, with the test set accounting for 30% and the training set accounting for 70%. Figure 7 depicts the prediction results of the two models.

The performance of both baseline methods in processing spectral data was unsatisfactory. The $R^{2}$ obtained with the linear regression model was only 0.37, and the RMSE was 0.80. The random forest model achieved slightly more accurate results, with an $R^{2}$ of 0.50 and an RMSE of 0.78. These results required improvement. To explore the effectiveness of the proposed SVM algorithm model, we directly modeled the original data using SVM and compared it with RF and LR. The comparison results are shown in Table 2.

The experimental findings revealed that the SVM algorithm outperformed the other two algorithms and the traditional approach according to the correlation coefficient on both the test and prediction sets. Although SVM’s performance on the training set is not optimal, this does not show the true predictive power of SVM. In the evaluation of the prediction model, more attention should be paid to the model’s ability to process unknown data, and the SVM model has significantly better indicators in processing test set data than other methods, so the SVM model has more effective prediction ability than other methods. The LR algorithm obtained an exceptionally low correlation coefficient of zero due to overfitting, which did not imply that LR had high prediction ability. The prediction set’s root mean square error intuitively demonstrated SVM’s superiority in the prediction process compared with the other two classical techniques. These results indicated the SVM method’s capacity to cope with small samples, nonlinearity, and high dimensionality, as well as the success of modeling based on the SVM algorithm in this study. Below, we focus on the preprocessing, feature wavelength extraction, and support vector machine model optimization of the hyperspectral sugar content prediction algorithm in detail.

### 4.3. Data Processing Effectiveness

Spectral data must be preprocessed before formal modeling to reduce nonsystematic flaws such as instrument noise and dark current [44]. We used three techniques to generate support vector regression (SVR) to analyze the influence of several preprocessing methods: standard normal transformation (SNV), multivariate scattering correction (MSC), and convolution smoothing (SG). Table 3 describes the model and its prediction effect.

**Figure 8:**
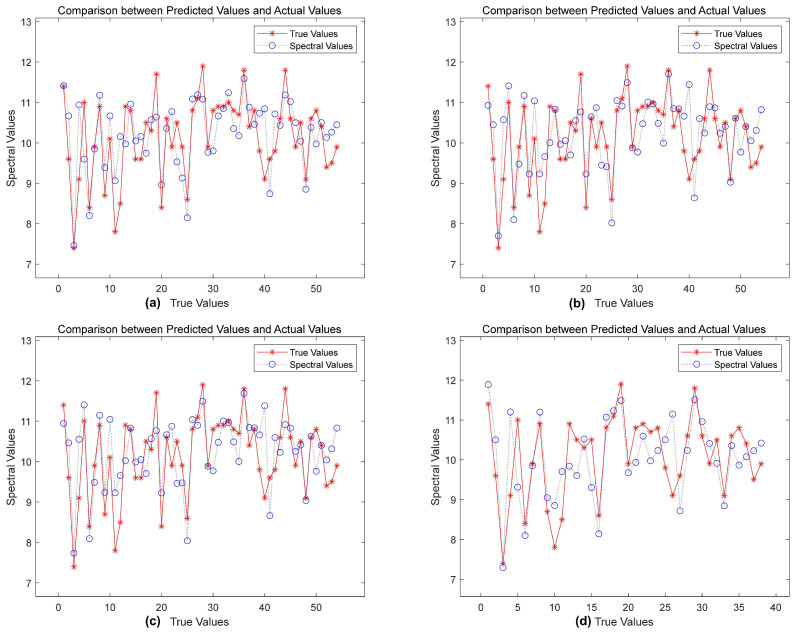
Line chart of predicted and true values under different data processing techniques: (**a**) the prediction result of the original data; the hyperspectral data after (**b**) convolution smoothing and (**c**) standard normal transformation. (**b**) The line chart corresponding to the standard normal transformation data; the prediction map corresponding to the (**c**) multivariate scattering correction method and (**d**) convolution smoothing method.

The evaluation indicators in Table 3 show that, compared with the baseline methods shown in Figure 4, the correlation coefficient of the preprocessed data was higher and the root mean square error was smaller. Compared with the baseline methods, substantial performance improvements were achieved after the original data were modeled using SVR ($R_{p}^{2}=0.7093,\;RMSEP=0.5619$). Additionally, when the original data were subjected to the three preprocessing technologies of SNV, MSC, and SG, the effect was further strengthened, the correlation coefficient index was increased, and the root mean square error was reduced. The performance of SG–SVR was particularly notable ($R_{p}^{2}=0.7835,\;RMSEP=0.4442$). We provide a line chart of predicted and true values in Figure 8 to present these results.

The SVR model generated using the original data was near to the true value in the early data stages, as shown in the upper left of Figure 8, but, when the genuine value changed little, its prediction accuracy was considerably reduced. The SVR model constructed with SG-preprocessed data achieved robust prediction performance closer to the true value. This demonstrates how SG preprocessing technology can increase data quality and, thus, the model’s robustness and detection accuracy.

**Figure 9:**
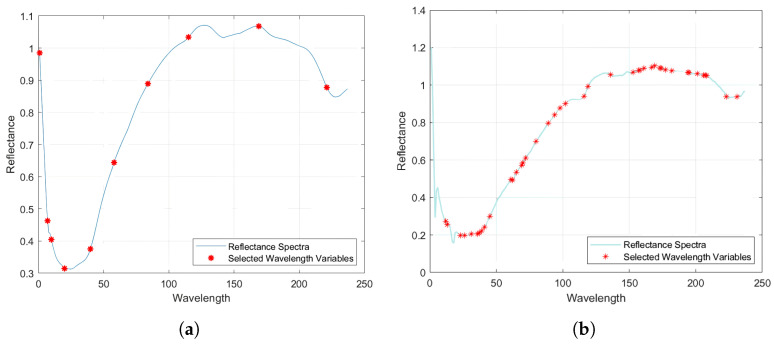
Two types of feature band extraction algorithms were used to extract band differences: the extraction result of (**a**) CARS algorithm and (**b**) SPA.

Although preprocessing can successfully reduce the impacts of noise and scattering on spectral data analysis, redundant and overlapping band data still exist in full-spectrum data [45]. Using full-band modeling not only results in computing inefficiencies, but also lowers the model’s prediction accuracy [46]. As a result, typical wavelength screening is necessary for full-spectrum data following SG preprocessing to minimize the dimension of the data and remove information that is unnecessary to the detection indications. This speeds up model training and increases forecast accuracy [47]. The competitive adaptive reweighting algorithm (CARS) and sequential projection algorithm (SPA) were employed in this study to detect the characteristic spectral wavelengths of the Brix of crisp pear. Figure 9 depicts the filtered characteristic wavelengths.

**Figure 10:**
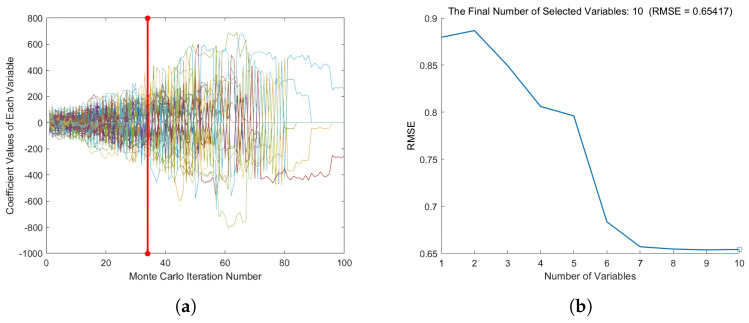
The regression coefficient path diagram during the CARS sampling operation (**a**) and the relationship between the number of SPA features and RMSE (**b**).

**Figure 11:**
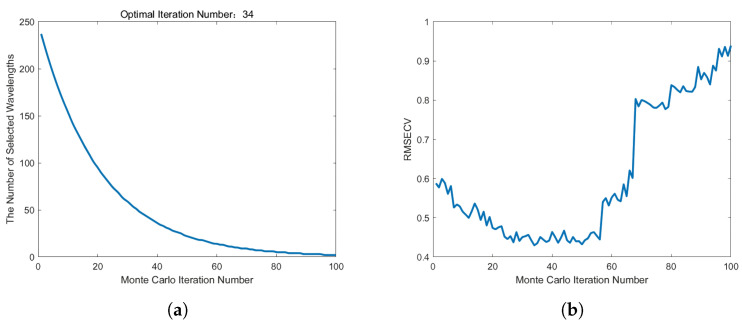
Characteristic wavelength screening results obtained with CARS. (**a**) Change in the number of CARS wavelengths as the number of iterations increases; (**b**) the change curve with increasing number of RMSECV iterations.

Figure 9 shows that 42 feature variables were retrieved after applying the CARS algorithm to extract features from the spectral data, accounting for 17% of the total number of hyperspectral variables. The retrieved characteristic bands were mostly centered within 40 nm, and the characteristic variable distribution was reasonably continuous. Figure 10 shows that the SPA yielded a total of 10 distinctive bands, accounting for 4% of the total number of hyperspectral spectra. Its distinctive bands were primarily concentrated within 50 nm, with a relatively high frequency of characteristic bands occurring at the trough. Figure 11 depicts typical results of wavelength screening using the CARS method. The number of Monte Carlo samples in this approach was set to 50, the cross-validation number was set to 10, and the ideal principal component number was set to 10. Figure 11 depicts how the number of distinctive wavelengths changes as the number of Monte Carlo sampling iterations rises. Figure 11 shows that, when the number of iterations rose, the root mean square error cross-validation (RMSECV) of each subset changed. The early phases of this transition indicated that the model’s prediction error gradually decreased via the deletion of redundant and irrelevant information. However, as the iterations progressed, the inaccuracy increased, which may have been due to over-screening and picking too many features.

Figure 10 depicts the path map of the variable’s regression coefficient during the sampling operation. In Figure 10a, the prominent red vertical line properly identifies the minimum RMSECV, as well as the best subsection, chosen with the CARS algorithm. The dimensionality of data can be reduced by projecting them step-by-step to discover the most important features using the sequential projection algorithm (SPA). In this study, we used SPA to screen out the characteristic wavelengths that were most important to the prediction aim from 237 wavelengths of the sugar content spectrum. Figure 10b depicts the link between the number of features and the root mean square error (RMSE). The RMSE rapidly lowered as the number of selected characteristics rose, as shown in Figure 10. This means that, as more relevant features were incorporated into the model, the prediction accuracy was further increased.

We created an SVM model using feature data and compared the results obtained on the training and prediction sets with those of two alternative feature extraction strategies, as shown in Table 4. Table 4 shows that the prediction set performance of the SVM model constructed using the two feature wavelength extraction approaches was quite similar, with CARS performing relatively well. The CARS algorithm’s optimal point for feature extraction, RMSECV, was 0.4550, the number of iterations was 34, and 42 essential feature wavelengths were successfully screened out. SPA effectively screened out the 10 most representative distinctive wavelengths from the original 237 wavelengths when the RMSE reached the lowest threshold of 0.6542. Despite SPA filtering fewer and lighter feature wavelengths, the indicators were inferior to those extracted with CARS. The cause for this is that, during the feature-screening phase, the SPA deleted too many features, resulting in the remaining wavelengths being insufficient for accurately reproducing the original spectral properties. Because the SPA performed poorly in screening higher wavelengths, we applied CARS in the following tests to extract distinctive wavelengths.

### 4.4. Optimization Algorithm Effectiveness

**Figure 12:**
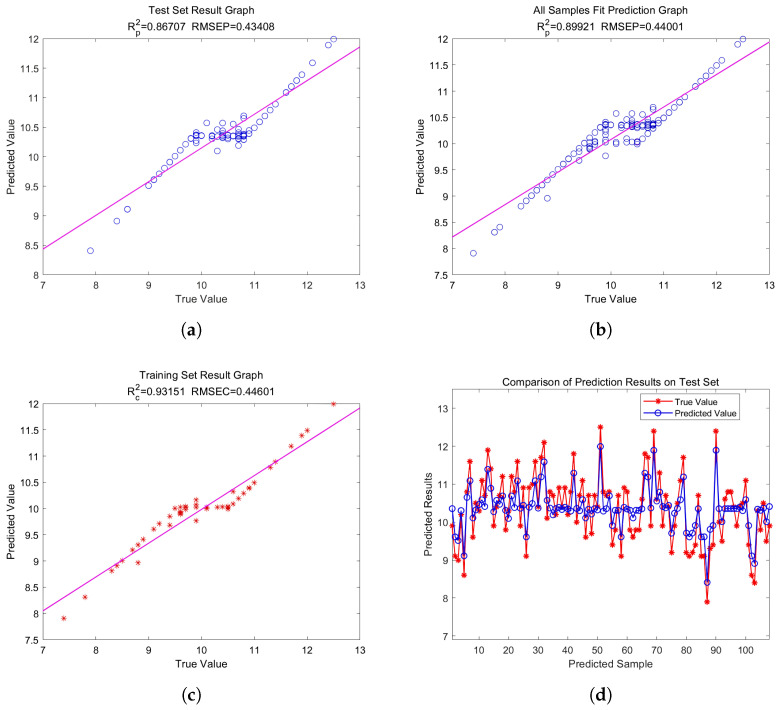
The final model prediction effect. The blue circle and red asterisk in the figure represent the sample points, and the curve in the figure represents the fitting under the corresponding sample points. (**a**) Test set result graph (performance measurement graph); (**b**) All samples fit prediction graph (test set performance measurement graph); (**c**) Training set result graph (training set performance measurement scatter plot of CARS–GASVR model); (**d**) Comparison of prediction results on test set (test value and true fitted plot of values).

(1) Performance comparison of SVR and GASVR models
  The SG-preprocessed full-wavelength data were used to build a genetic-algorithm-optimized support vector machine regression (GASVR) model. The outcomes of this method were compared with those from the classic support vector machine regression (SVR) model. Table 5 displays the specific outcomes.
  The results in Table 4 show that, compared with the SVR model, GASVR exhibited stabler and superior fitting on the training set ($R_{c}^{2}=0.8945,\;RMSEC=0.4709$). Specifically, the determination coefficient ($R_{p}^{2}=0.8156$) on the prediction set was 0.0321 higher than that of the SVR model ($R_{p}^{2}=0.7835$). The root mean square error on the prediction set was 0.0267 lower than that of the SVR model (RMSEP=0.4442). This verified that optimization using the genetic algorithm substantially improved the performance of the support vector regression prediction model;
(2) Construction of GASVR regression model
  To find the best model for predicting crisp pear sugar content, we built a GASVR model using the 48 and 10 distinctive wavelengths screened using CARS and SPA, respectively. We then used the full-wavelength GASVR model prediction findings as the reference standard. Table 6 shows the prediction impacts of the GASVR model for these three different preprocessed wavelength inputs.
  Table 6 shows that most of the final performance indicators of the GASVR model established using the wavelengths processed via feature engineering were better than those of the GASVR model established using the original wavelengths. The final performance index of the GASVR model established after CARS characteristic wavelength extraction was generally higher than that of the model established after SPA characteristic wavelength extraction. Therefore, the optimal model was the SG-CARS-GASVR model established using the characteristic wavelengths filtered with CARS, which achieved an $R^{2}=0.8992\;\mathrm{and}\;\mathrm{an}\;RMSE=0.4400$. Ranked second was the MSC–CARS–GASVR model, established using the characteristic wavelengths filtered with CARS, which achieved an $R^{2}=0.8812\;\mathrm{and}\;\mathrm{an}\;RMSE=0.4310$. The results on the calibration set ($R^{2}=0.8550\;\mathrm{and}\;RMSE=0.4709$ ) were substantially improved compared with those of the full-wavelength model. To summarize, the GASVR model produced more accurate predictions, and the SG–CARS–GASVR model produced the best prediction performance overall. Scatter plots of the overall evaluation of the CARS–GASVR model, the test set evaluation, the training set evaluation, and the fitting diagrams of the test and true values in the test set are shown in Figure 12.

We developed a method for estimating pear sugar content based on a support vector machine (SVM), and we optimized the model using the genetic algorithm (GA), with the goal of increasing prediction accuracy. We also conducted a comparison of our results with those obtained in other studies, as shown in Table 7.

## 5. Conclusions

We developed a method for estimating the sugar content in pears using a support vector machine (SVM). We optimized the model using the genetic algorithm (GA), with the goal of increasing the accuracy of sugar content prediction. First, crisp pear spectral data were subjected to extensive preprocessing, including standard normal transformation (SNV), multivariate scattering correction (MSC), and convolution smoothing (SG). The SG technique produced the best results in the preprocessing stage. Finally, the competitive adaptive reweighting (CARS) method and the continuous projection algorithm (SPA) were used to screen the characteristic wavelengths, and an optimized GA support vector machine (GASVR) model was built on this basis.

According to the findings, the GASVR model increased the prediction set correlation coefficient by 0.0321 compared with that of the classic SVM model, resulting in higher prediction accuracy. The GASVR model also reduced the root mean square error on the prediction set by 0.0267 compared with that of the SVR model. The CARS approach chose 48 distinctive wavelengths, whereas the SPA method chose 10 important wavelengths throughout the feature selection process. Both methods outperformed the full-wavelength model, demonstrating the efficiency of the characteristic wavelength selection approach. The CARS-based GASVR model performed exceptionally well. Finally, the C (3.22) and g (0.51) parameters of the support vector machine optimized using the genetic method were determined. Compared with the full-wavelength GASVR model, the optimized model’s coefficient of determination was 0.0442 higher, and the root mean square error was 0.0309 lower. As a result, we illustrated the effectiveness of employing a genetic algorithm to refine SVM to create a crisp pear sugar content prediction model.

In the future, we plan to add noise to the original data for training during the preprocessing stage to improve the model’s generalization ability; additional quantitative research will also be conducted on the optimization of model parameters to investigate the specific impact of these parameters on model training. Given the possibility of using more kinds of fruit and sugar content ranges in practical applications, more thorough empirical studies and verification under varied environmental conditions will be important aspects of future study.

## Figures and Tables

**Table 1:** The training set and the dataset were divided according to a ratio of 118:50; the distribution of the data under different criteria is summarized.

	Dataset	Sample Size	Minimum Value	Maximum Value	Average Value	(Statistics) Standard Deviation
Hyperspectral sample set	Training set	118	7.4	12.5	10.5	2.83
Segmentation	Test set	50	8.4	10.5	9.7	1.52

**Table 2:** The performance of three benchmark models under different test indexes is compared.

Preprocessing	$R_{c}^{2}$	*RMSEC*	$R_{p}^{2}$	*RMSEP*
Support Vector Machine	**0.6985**	**0.6358**	**0.7093**	**0.5619**
Random Forest	0.8986	0.3648	0.5047	0.7832
Linear Regression	0.1050	0.1579	0.3703	0.7975

**Table 3:** The performance of three pretreatment methods compared with the original data under the SVM model is compared.

Preprocessing	$R_{c}^{2}$	*RMSEC*	$R_{p}^{2}$	*RMSEP*
Raw Data	0.6985	0.6358	0.7093	0.5619
Standard Normal Transformation	0.7163	0.5288	0.7573	0.4490
Multivariate Scattering Correction	0.7114	0.5562	0.7402	0.4793
Convolution Smoothing	**0.7475**	**0.5212**	**0.7835**	**0.4442**

**Table 4:** The performance of two feature extraction methods after SG preprocessing under SVM model is compared.

Feature Extraction Method	Number of Characteristic Variables	*RMSEC*	$R_{p}^{2}$	*RMSEP*
**CARS**	**42**	**0.4550**	**0.7433**	**0.6912**
SPA	10	0.6542	0.7278	0.6915

**Table 5:** The performance of SVR model before and after GA optimization is compared.

Pretreatment	Developed Model	Training Set		Test Set	
		$R_{c}^{2}$	***RMSEC***	$R_{p}^{2}$	***RMSEP***
SG	SVR	0.7475	0.5212	0.7835	0.4442
**SG**	**GASVR**	**0.8945**	**0.4067**	**0.8156**	**0.4175**

**Table 6:** GA–SVR modeling result.

Pretreatment		Optimal Parameters C, g	Model Developed	Final $R^{2}$	*RMSE*
Raw Data		2.8/0.13	GASVR	0.8550	0.4709
SNV	CARS	3.22/0.51		0.8774	0.4287
MSC				0.8812	0.4310
SG				0.8992	0.4400
SNV	SPA	7.83/1.38		0.6259	0.6203
MSC				0.8705	0.4226
SG				0.8409	0.4428

**Table 7:** Comparison of results among the proposed method and previously reported methods.

Author	Study Target	Model	$R^{2}$
Ours	Sugar content in crispy pears	SG–CARS–GASVR	0.8992
Wei et al. [48]	Persimmon Brix	Savitzky–Golay– RS–CARS–PLS	0.757
Kim et al. [49]	Soluble solids content in citrus fruits	CARS–PLSR	0.75
Zhao, Yu, and He [50]	Total soluble solids in mulberry	LS–SVM–linear	0.857

## Data Availability

Data are contained within the article.
